# A Fine Morphological Study of the Rare *Anillidris bruchi* Santschi (Hymenoptera: Formicidae: Dolichoderinae) Male and Queen

**DOI:** 10.3390/insects14090723

**Published:** 2023-08-23

**Authors:** Stefano Cantone, Andrea Di Giulio

**Affiliations:** 1 Department of Science, University Roma Tre, 00146 Rome, Italy; 2 Laboratorio Interdipartimentale di Microscopia Elettronica (LIME), University Roma Tre, 00146 Rome, Italy; 3 National Biodiversity Future Center (NBFC), 90133 Palermo, Italy

**Keywords:** winged ants, external genitalia, ant spurs, SEM, ant morphology, volsella sensorium

## Abstract

**Simple Summary:**

In this study, based on specimens collected in the city of São Paulo (Brazil), we redescribe and illustrate the rare Neotropical ant *Anillidris bruchi* male and queen by using optical and scanning electron microscopy (SEM), supplying new morphological distinctive characters, and improving the previous descriptions. This work will be useful for identifying the alate castes of this species and for using the characters of the male and queen to infer relationships with other dolichoderine ants.

**Abstract:**

Using optical and scanning electron microscopy, we describe the following new morphologically distinctive characters of the rare Neotropical ant *Anillidris bruchi* Santschi, 1936, male and queen: scattered setae inter-ommatidia, semicircular hypostomal notch, antennal cleaning, metatibial spurs, and the remnant of the M2 vein in the hindwings. In males, we show for the first time the morphology of maxillary and labial palpi, the absence of metapleural glands, and, in external genitalia, for the first time in ants, a new mechano-sensory area on the volsella that we called “volsella sensorium”, composed of several spine-like sensilla. Additionally, we give an updated morphological diagnosis of the alate caste, which will be useful for future studies to clarify the phylogeny of the genus *Anillidris*.

## 1. Introduction

The dolichoderine ant genus *Anillidris* is known by a single species, *Anillidris bruchi* Santschi, 1936. Due to its strict subterranean habit [1,2,3] the species was very rarely collected; for this reason, only a few specimens of *A. bruchi* are deposited in entomological collections, and only scattered information is available. 

The species was described based on workers collected in Loreto, Missiones (Argentina) [1]. Later, Santschi [4] described the winged castes (male and queen) of *A. bruchi*, providing diagnostic characters for morphological identification. In the same paper, the author suggested that, similar to parasitic ants with tiny workers and larger queens, the species may show lestobiotic behavior [4]. Borgmeier [5] mentioned workers, males, and queens of *A. bruchi* collected in Nova Teutônia, Santa Catarina (Brazil), and Shattuck [2] redescribed all castes of *A. bruchi* in a revision of the Dolichoderinae genera. Later, Schmidt et al. [3] captured *A. bruchi* workers in Viçosa, Minas Gerais (Brazil), using hypogaeic pitfall traps, showing, for the first time, pictures of the habitus and head of the male, queen, and worker. Additionally, Schmidt et al. [3] reported on a queen captured in 1961 in Cotia, São Paulo, Brazil, and deposited in the Museum of Zoology of the University of São Paulo. The last presence was recorded in 2012–2013 by Cantone [6,7,8], who collected queens and males of *A. bruchi* in São Paulo city and presented a brief diagnosis of the genus *Anillidris* male and queen; finally, Cantone and Von Zuben [9] showed the hindwing of *A. bruchi* male. 

In the present study, based on specimens collected in the city of São Paulo, we redescribe and illustrate *A. bruchi* male and queen by using optical and scanning electron microscopy (SEM), supplying new morphological distinctive characters and improving both the previous descriptions by Santschi [4] and Shattuck [2] and the brief diagnoses of male and queen by Cantone [6,7]. 

## 2. Materials and Methods

Field collecting was conducted in the city of São Paulo (Brazil), 23°35′16.13″ S, 46°38′55.02″ W, altitude 800 m a.s.l., and 23°27′33.41″ S, 46°38′17.10″ W, altitude 800 m above sea level. Specimens were captured by using two light traps of the “Luiz de Queiroz” model, equipped with 15 watts UV black blue lamps. The traps were left in the same position, attached to the same tree at 3 m and 7 m from the ground, kept active continuously, and checked weekly [8]. Three winged queens and five winged males of *A. bruchi* were collected by S. Cantone from September to November 2012 and September and October 2013 (collecting permit license SISBIO 40144-2). Voucher specimens are deposited in the Museum of the Department of Science, University Roma Tre (Rome, Italy). The identification of the male and queen of *A. bruchi* was performed using descriptions by Santschi [4] and the diagnosis by Shattuck [2] and Cantone [6,7]. 

### 2.1. Morphological Analysis

Dissections were performed under an Olympus SZX16 (Olympus, Tokyo, Japan) stereo microscope equipped with Olympus KL1500 LCD fiber optics (Olympus, Tokyo, Japan). Photographs were acquired using an Axiocam 503 (Carl Zeiss Microimaging Gmbh, Jena, Germany) mounted on a Zeiss AxioZoom V16 (Carl Zeiss AG; Oberkochen, Germany) with LED dual spotlights from Photonic Optische (Vienna, Austria). The SEM (scanning electron microscope) analysis was performed at the L.I.M.E. laboratory (University of Roma Tre, Rome, Italy). Samples were gradually dehydrated in ethanol series (70%, 85%, 95%, 30 min each, and 100% for 2 h), critical point-dried (Balzer Union CPD 030 unit), mounted on aluminum stubs by self-adhesive conductive carbon disks, gold sputtered with an Emithech K550 sputter coater (Emithech, Kent, UK), and analyzed with a Zeiss Gemini 300 field emission SEM microscope (Carl Zeiss AG, Jena, Germany) using a voltage of 5 kV.

Measurements are based on a single male and a single queen specimen and were taken using an SEM microscope on dissected specimens.

The morphological standards and the terminology used in this work follow Cantone and Di Giulio [10].

### 2.2. Measurements

HL: head length, in full face view. The midline distance from the level of the maximum posterior projection of the margin of the head (not including the ocelli) to the level of the most anterior projection of the anterior clypeal margin. 

HW: head width, in full face view, the maximum width of the head posterior to the compound eyes. SL: antennal scape length, measured from the apex of the first antennal segment to the base, exclusive of the radicle. EL: eye length, in lateral view, the length of the compound eye along the longitudinal axis. EW: eye width, in lateral view, the maximum transverse width of the compound eye. MML: maximum mesosomal length in lateral view from the anteromedial part of the pronotum to the posteromedial part of the propodeum. WL: forewing length, the maximum distance between the insertion of the sclerotized wing veins and the distal margin of the wing. WHL: hindwing length, the maximum distance between the insertion of the sclerotized wing veins and the distal margin of the wing. CI: cephalic index, 100x HW/HL. SI: scape index, 100x SL/HL. OI: ocular index, 100x EL/HL. WI: wing index, 10x WL/MML. ES: eye size, 100x ELxEW.

## 3. Results

Genus Anillidris Santschi, 1936
Species Anillidris bruchi Santschi, 1936
(Figure 1, Figure 2, Figure 3, Figure 4, Figure 5, Figure 6, Figure 7, Figure 8, Figure 9, Figure 10 and Figure 11)

**Male diagnosis**: Antennae filiform, with 13 articles; scape as long as the first two articles of the funiculus. Eyes with scattered setae inter-ommatidia. Mandibles triangular, and dentate. Maxillary palpi of three articles and labial palpi of four articles, with the fourth article partially fused with the previous one. Medial hypostoma with a distinctive, regular, semicircular notch. Mesonotum without notauli. Propodeum without teeth or spines. Metapleural gland orifice absent. Metatibiae with single short spur, triangular in shape, and broad at the base. Forewings with submarginal I and II cells, discoidal cell and marginal cell closed. Hindwings with remnants of M2 vein, nebulous distally. Pretarsal claws simple and large at the base. Petiole broadly articulated posteriorly with the abdominal segment III and with ventral process developed. Pygostyles present. External genitalia with basimere extending in a semicircular lobe ventrally, and telomere developed in lobe shape and being longer than the digitus posteriorly; digitus with apicodorsal process, spiniform apically and with lateral “volsella sensorium” (Figure 7A,B,D); cuspis and basivolsellar process reduced. 

**Queen diagnosis**: Antennae filiform with 12 articles, scape slightly overstepping the occiput. Eyes with scattered setae inter-ommatidia. Anteromedial part of clypeus straight in dorsal view. Mandibles triangular and dentate. Maxillary palpi of three articles elongated, and labial palpi of four short articles. Medial hypostoma with a distinctive regular semicircular notch. Propodeum without teeth or spines. Metapleural gland orifice present. Metatibiae with single short spur, as in male. Forewings and hindwings as in male. Petiole vertical with abundant setae laterally. Forth tergite of the gaster very developed, covering the fifth.

**Male**

Voucher material: Brazil, São Paulo city. Two specimens deposited in the Museum of Zoology of the Roma Tre University (Rome, Italy). 

Measurements (in mm): HL: 0.62; HW: 0.67; SL: 0.2; EL: 0.27; EW: 0.22; MML: 1.47; WL: 4.3; WHL: 3.3. 

Indices: CI: 108.06; SI: 32.26; OI: 43.55; WI: 29.25; ES: 5.94.

**Figure 1:**
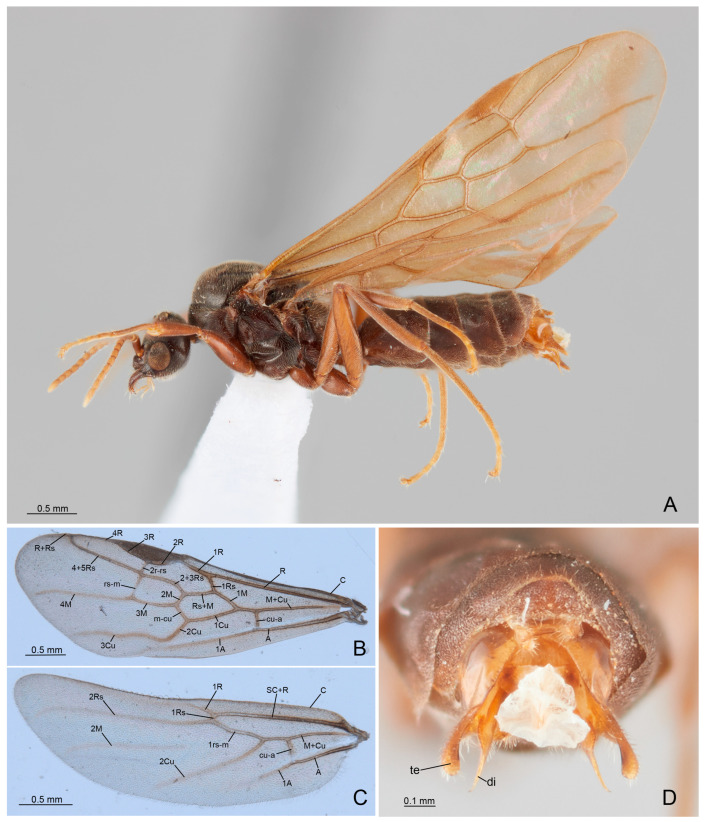
*Anillidris bruchi* male. (**A**) habitus; (**B**) forewing; (**C**) hindwing; and (**D**) external genitalia in posterior view. Abbreviations: A: Anal vein; C: Costal vein; Cu: Cubital vein; cu-a: cubital-anal cross-vein; di: digitus; M: Media vein; m-cu: media-cubtus cross vein; rs-m: radial sector-media cross vein; r-rs: radius-radial sector cross vein; R: Radius vein; Rs: Radial sector; SC: Subcostal vein; te: telomere.

**Figure 2:**
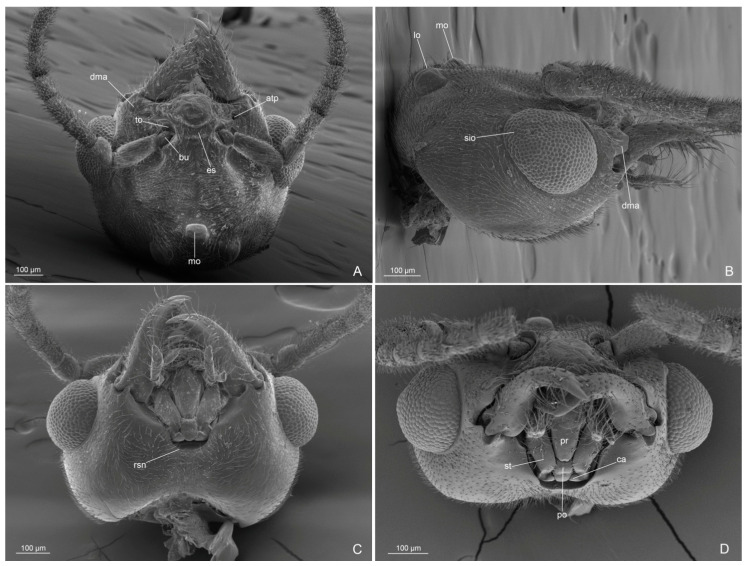
*Anillidris bruchi* male. (**A**) head in dorsal view; (**B**) head in lateral view; (**C**) head in ventral view; and (**D**) head in anterior view. Abbreviations: atp: anterior tentorial pit; bu: bulbus; ca: cardo; dma: dorsal clypeal mandible articulation; es: epistomal sulcus; lo: lateral ocellus; mo: median ocellus; po: postmentum; pr: prementum; rsn: regular semicircular notch; sio: setae inter-ommatidia; to: torulus.

**Figure 3:**
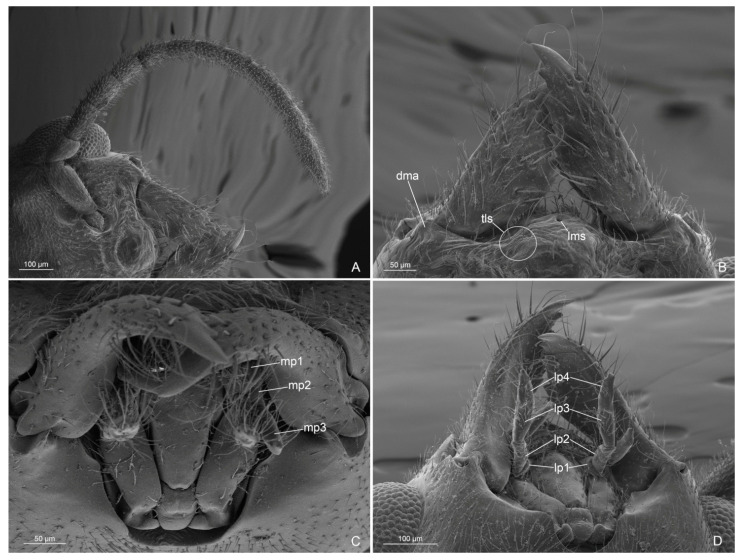
*Anillidris bruchi* male. (**A**) antenna; (**B**) mandibles in dorsal view; (**C**) maxillary palpi in ventro-anterior view; and (**D**) labial palpi in ventral view. Abbreviations: lms: long medial clypeal setae; lp: labial palpi; mp: maxillary palpi; tls: three long clypeal setae.

Head (Figure 1A,B, Figure 2A–D and Figure 3A–D): with abundant pubescence. Three ocelli present, with the median ocellus slightly smaller than the lateral ocelli. Eyes convex, slightly narrowing posteriorly, occupying the anterolateral side of the head; scattered setae inter-ommatidia only posteriorly present. Antennae of 13 articles; scape as long as the first and second articles of funiculus combined; articles of funiculus with the following shape and size: first shortest and globose; second cylindrical and shorter than the last article (the longest); third slightly shorter than second; fourth to eleventh subequal in length and diameter; and shorter than the previous ones. Clypeus convex medially, with a single long medial seta and three pairs of long lateral setae. Dorsal clypeal mandibular articulation developed, extending on the base of the mandible. Mandibles with long apical tooth, followed by three large teeth alternate with 3–4 denticles or small teeth. Medial hypostoma with a distinctive, regular, semicircular notch. Maxillary palpi of three articles, with the first article short; labial palpi of four articles; the fourth article fused partially with the previous one.

**Figure 4:**
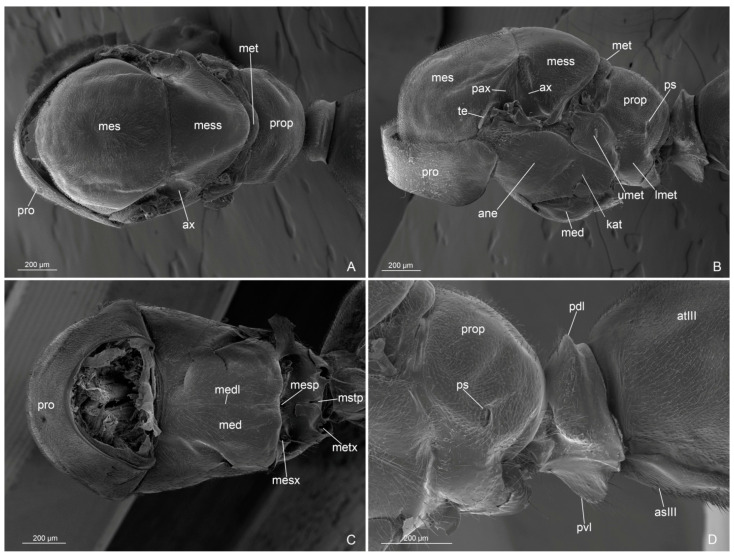
*Anillidris bruchi* male. (**A**) mesosoma in dorsal view; (**B**) mesosoma in lateral view; (**C**) mesosoma in ventral view; and (**D**) petiole in lateral view. Abbreviations: ane: anepisternum; as III: abdominal sternite III; at III: abdominal tergite III; ax: axilla; lmet: lower metapleuron; kat: katepisternum; med: mesodiscrimen; medl: mesodiscrimenal line; mes: mesoscutum; mesp: mesoprefurcal pit; mess: mesoscutellum; mesx: mesocoxal foramen; met: metascutellum; metx: metacoxal foramen; pax: preaxilla; pdl: petiolar dorsal lobe; pro: pronotum; prop: propodeum; ps: propodeal spiracle; pvl: petiolar ventral lobe; te: tegula; umet: upper metapleuron.

**Figure 5:**
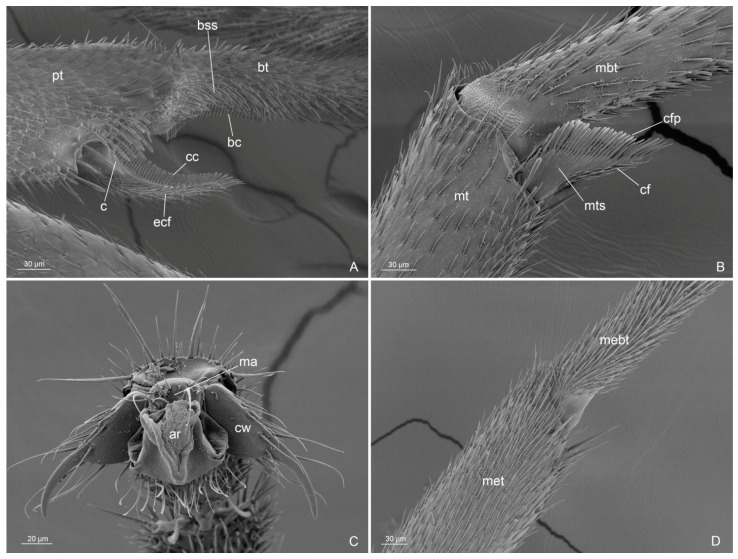
*Anillidris bruchi* male. (**A**) antennal cleaning; (**B**) metatibial spur; (**C**) pretarsal claw; and (**D**) mesotibiae without spur. Abbreviations: ar: arolium; bc: basitarsal comb; bss: basitarsal spatulate setae; bt: probasitarsal; cc: calcar comb; cf: cuticular fringes; cfp: cuticular fringes pectinate; cw: claw; ecf: external cuticular fringes; ma: manubrium; mbt: metabasitarsal; met: mesotibiae; mebt: mesobasitarsal mt: metatibiae; mts: metatibiae spur; pt: protibiae.

Mesosoma (Figure 1C,D, Figure 4A–D and Figure 5A–D): Pronotum short, with abundant decumbent pubescence. Mesoscutum in lateral view strongly convex anteriorly, not overhanging the pronotum, and covered by decumbent pubescence dorsally and laterally; notauli absent; parapsidial lines evident. Mesoscutellum convex and slightly higher than mesoscutum, with scarce pubescence dorsally. Scutoscutellar sulcus absent. Metascutellum convex dorsally, lower than mesoscutellum and not overlapping the propodeum. Mesodiscrimen ventrally very developed, covering the anterior margin of the mesocoxal foramen. Metapleural gland orifice absent (no glandular openings visible). Metapleural suture between lower metapleuron and propodeum absent. Propodeum convex, in lateral view, with a dorsal declivous face longer than posterior face and with lateral pubescence and dorsal setae; angle of propodeum indistinct; spiracular orifice lateral and vertical. Forewings with submarginal 1 and 2 cells, discoidal cell with Media 2 present, marginal cell closed; pterostigma dark; 2 radius-radial sector cross-vein in line with radial sector-media; radial sector+media crossvein nebulous in central part. Hindwings with Media 2 vein absent, but with vein remnants, nebulous distally; 11–15 hamuli, 1 Radius vein nebulous and 1 Radial sector present and short. The forewing venation variation is shown in Figure S1 (Supplementary Material) with extra cross-vein in discoidal cell. Antennal cleaning with protibiae calcar short, broad, with comb in the inner margin and cuticular decumbent fringes in the outer margin; probasitarsus with inner comb and outer spatulate setae. Mesotibiae without spurs. Metatibiae with single short spur, triangular in shape and broad at base; inner margin with very long cuticular pectinate fringes and outer margin with long decumbent cuticular fringes. Pretarsal claw simple and very large at the base.

**Figure 6:**
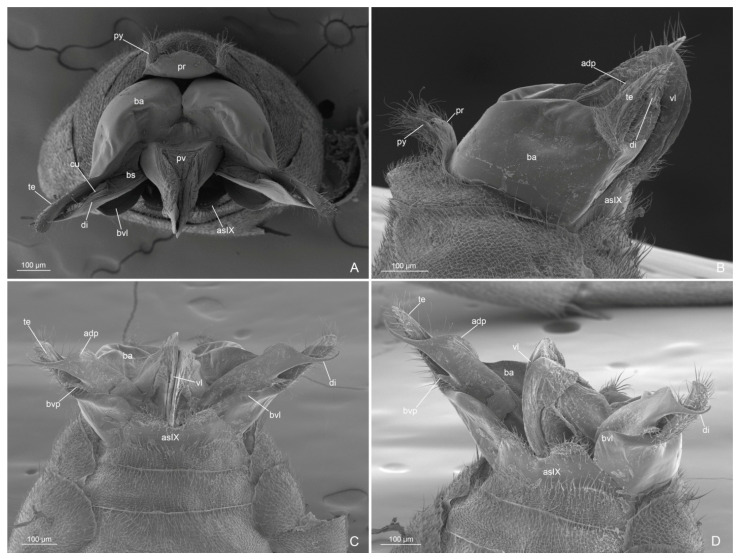
*Anillidris bruchi* male. (**A**) external genitalia in posterior view; (**B**) external genitalia in lateral view; (**C**) external genitalia in ventral view; and (**D**) external genitalia in ventro-lateral view. Abbreviations: adp: apicodorsal process of the digitus; as IX: abdominal sterinite IX; pr: proctiger; ba: basimere; bs: basivolsella; bvl: basimere ventral lobe; bvp: basivolsellar process; cu: cuspis; di: digitus; pv: penis valve; py: pygostyle; te: telomere; vl: valviceps lamina.

**Figure 7:**
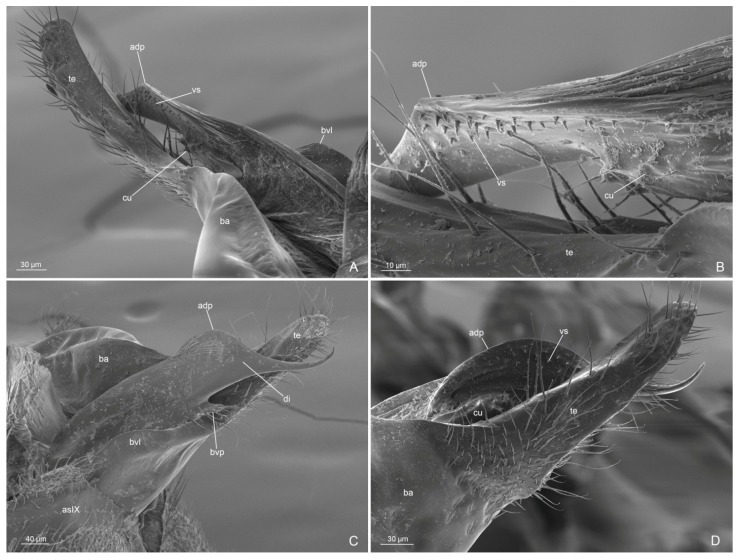
*Anillidris bruchi* male. (**A**,**B**,**D**) paramere and volsella in dorsal view; and (**C**) paramere and volsella in ventro-mesal view. Abbreviations: adp: apicodorsal process of the digitus; as IX: abdominal sterinite IX; ba: basimere; bvl: basimere ventral lobe; bvp: basivolsellar process; cu: cuspis; vs: volsella sensorium; te: telomere.

Metasoma (Figure 4D, Figure 6A–D and Figure 7A–D): petiole broadly articulated posteriorly, with entire anterior surface of abdominal segment III; in lateral view, a large ventral process, posteriorly directed, and a small dorsal lobe present; anterolateral pubescence decumbent and anterodorsal setae erected; posterolateral face without pubescence and setae. Pygostyles present. Proctiger short. 

External genitalia: posterior margin of the abdominal sternite IX with a large notch, semicircular medially and bilobed laterally. Paramere with basimere very developed dorsally and laterally, extending in a semicircular lobe ventrally; telomere developed in lobe shape, longer than digitus posteriorly. Volsella with digitus very developed, presenting an apicodorsal process with spiniform sensilla in lateral surface, representing the “volsella sensorium”, ventrally straight and spiniform apically; parossiculus with cuspis reduced laterally in small lobe, with very short apical setae and basivolsellar process ventral, reduced, with setae ventrally and apically. Penisvalve with valviceps lamina without teeth, but with ondulate profile in lateral view.

**Queen**

Voucher material: Brazil, São Paulo city. Two specimens were deposited in the Museum of Zoology of the Roma Tre University (Rome, Italy).

Measurements (in mm): HL: 0.8; HW: 0.9; SL: 0.6; EL: 0.31; EW: 0.24; MML: 2.4; WL: 7.5; and WHL: 5.4. 

Indices: CI: 112.5; SI: 75; OI: 38.75; WI: 31.25; and ES: 7.44.

**Figure 8:**
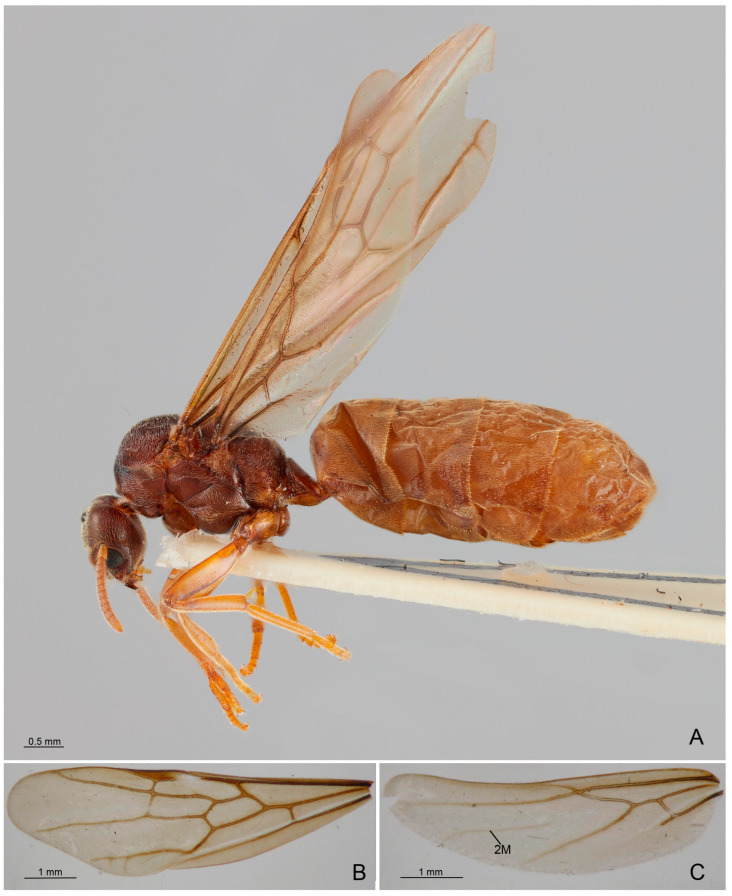
*Anillidris bruchi* queen. (**A**) habitus; (**B**) forewing; and (**C**) hindwing abbreviations: 2M: Media 2 vein.

**Figure 9:**
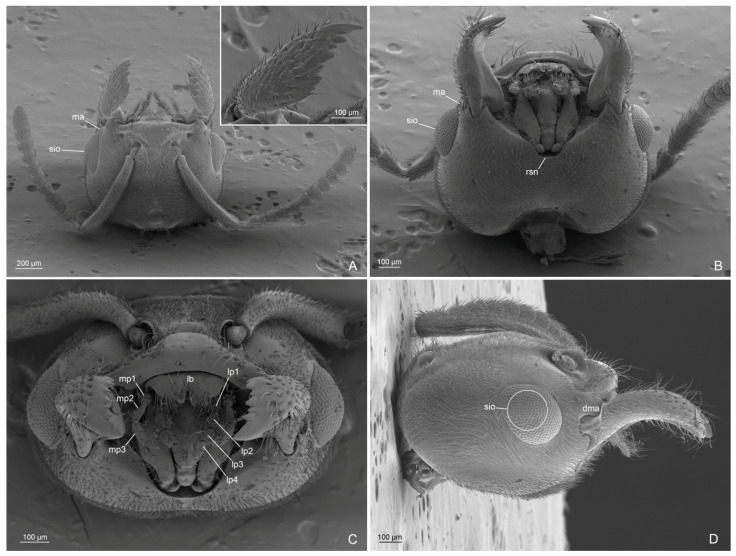
*Anillidris bruchi* queen. (**A**) head dorsal view; (**B**) head ventral view; (**C**) head anterior view; and (**D**) head lateral view. Abbreviations: dma: dorsal clypeal mandibular articulation; ma: malar area; lb: labrum; lp: labial palpi; mp: maxillar palpi; rsn: regular semicircular notch; sio: setae intra-ommatidia.

Head (Figure 8A and Figure 9A–D): with abundant decumbent pubescence. Three ocelli present. Eyes in the anterolateral side of the head convex, slightly narrower posteriorly, with scattered setae intra-ommatidia only posteriorly. Short malar area with long setae. Antennae with 12 articles; scape long, slightly overstepping the occiput; first article of the funiculus as long as the following two; articles III-X of funiculus subequal in length but gradually swelling towards the apex without forming a club; last article as long as the previous two articles together. Anteromedial part of clypeus straight in dorsal view, with about 20 setae; dorsal clypeal mandibular articulation developed. Mandibles with eight teeth: apical tooth longest, twice as long as subapical; five teeth and two small teeth at the base of the masticatory margin. Labrum bilobed with apical long setae. Medial hypostoma with a distinctive regular semicircular medial notch. Maxillary palpi of three articles, with first short, second and third longer and subequal in length; labial palpi of four articles, first three in barrel form, last article thinner.

**Figure 10:**
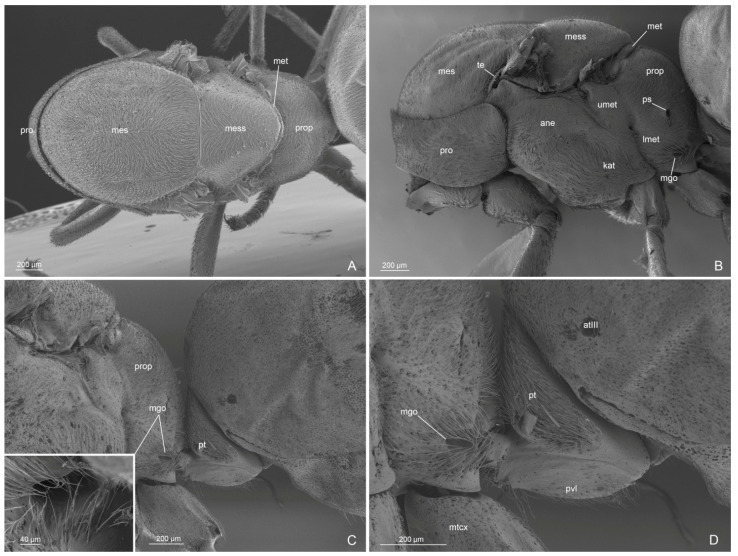
*Anillidris bruchi* queen. (**A**) mesosoma in dorsal view; (**B**) mesosoma in lateral view; (**C**) metapleural gland orifice; and (**D**) petiole in lateral view. Abbreviations: ane: anepisternum; at III: abdominal tergite III; kat: katepisternum; lmet: lower metapleuron; mtcx: metacoxe; mes: mesoscutum; mess: mesoscutellum; met: metascutellum; mgo: metapleural gland orifice; pro: pronotum; prop: propodeum; ps: propodeal spiracle; pt: petiole; pvl: petiolar ventral lobe; umet: upper metapleuron.

**Figure 11:**
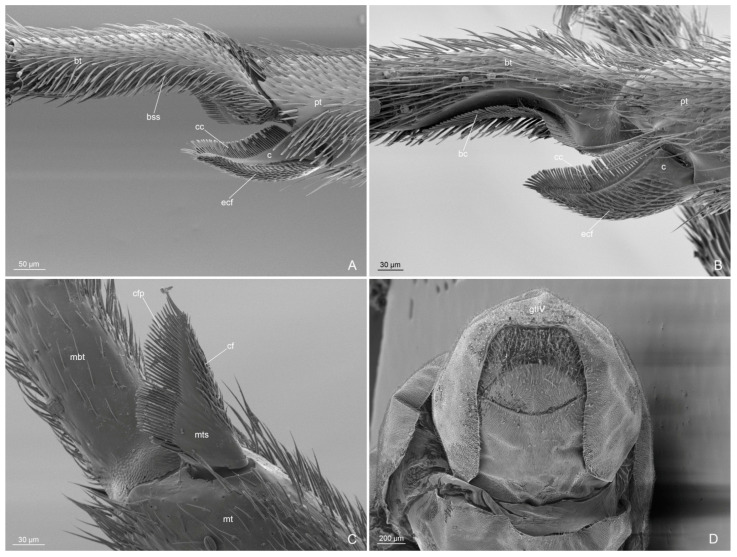
*Anillidris bruchi* queen. (**A**) antennal cleaning in anterior view; (**B**) antennal cleaning in posterior view; (**C**) metatibiae spur; and (**D**) last abdominal tergites and sternites in posterior view. Abbreviations: bc: basitarsal comb; bss: basitarsal spatulate setae; bt: probasitarsal; c: calcar; cc: calcar comb; cf: cuticular fringes; cfp: cuticular fringes pectinate; ecf: external cuticular fringes; gtIV: gastral tergite IV; mbt: metabasitarsal; mt: metatibiae; mts: metatibiae spur; pt: protibiae.

Mesosoma (Figure 8A–C, Figure 10A–D and Figure 11A–C): Pronotum with abundant decumbent pubescence. Mesoscutum strongly convex in lateral view, not overhanging the pronotum, totally covered by decumbent pubescence. Mesoscutellum about the same height as the mesoscutum. Metascutellum narrow and lower than mesoscutellum. Propodeum with dorsal declivous face shorter than posterior face and with abundant pubescence; angle indistinct; propodeal spiracle orifice lateral and rounded. Metapleural gland orifice present, with long setae on the edge. Metapleural suture between lower metapleuron and propodeum absent. Forewings with submarginal 1 and 2 cells and discoidal cell; marginal cell closed; pterostigma dark; Media 2 vein present; 2radius-radial sector cross-vein about in line with radial sector-media cross-vein; radial sector cross-vein nebulous in central part and in line with Media 1 vein. Hindwings with Media 2 vein absent, but with nebulous vein remnants distally; 1 Radius vein nebulous in part; radial sector absent; 16–17 hamuli. The forewing venation variation is shown in Figure S1 (Supplementary Material) with extra cross-vein in discoidal cell. Antennal cleaning structure as in males. Metatibiae with short and broad spurs with same structure as in male. Pretarsal claws as in male.

Metasoma (Figure 8A, Figure 10C,D and Figure 11D): Petiole with lateral surface with abundant long decumbent setae; posterior surface slightly concave without setae; anterior surface slightly convex with decumbent setae; ventral process slightly convex in lateral view with short posteroventral setae and long anteroventral setae. The fourth tergite of the gaster squared posteriorly, extending laterally on the sternite; and covering the fifth tergite.

## 4. Discussion

The genus *Anillidris* was placed by Santschi [4] near the genera *Dolichoderus* and *Leptomyrmex* for the morphology of the proventriculus and the forewing venation of winged castes. The relationships of the genus *Anillidris* within the Dolichoderinae are still unclear since *A. bruchi* was not available for sequencing in the most recent phylogenetic analysis of the subfamily based on molecular data [11]. However, the authors tentatively assigned this genus to the tribe Leptomyrmecini based on a few morphological similarities [11]. This paper is aimed at contributing to a better knowledge of the genus *Anillidris* by describing, through the use of SEM and optical microscopy, new fine morphological characters that could be useful in future phylogenetic analyses. Below, we list and discuss the most relevant characters here described for the first time in *A. bruchi*: 

(a) Eyes. In males and queens, the eyes are slightly narrower posteriorly with scattered setae inter-ommatidia posteriorly (Figure 2B and Figure 9A–D);

(b) Medial hypostoma. In males and queens, the medial hypostoma shows a distinct semicircular notch (Figure 2C and Figure 9B);

(c) Male palpi. We supply the first description of the male palpi since the palp formula reported by Shattuck [2] was erroneously referred to Santschi [1]. In fact, Santschi [1] described only the palpi of the worker (maxillary palpi of two articles and labial palpi of three articles). The same author [4] later described and illustrated those of the queen. The SEM photos that we supply of the queen palpi are consistent with the description by Santschi [4] (Figure 9D). Here we show that males have the maxillary palpi with three articles (Figure 3C) and the labial palpi with four articles, with the third and fourth articles partially fused (Figure 3D);

(d) Antennal cleaning. This is the first description of the antennal cleaning that shows the same structure in males and queens, with a short protibial calcar (Figure 5A and Figure 11A,B); 

(e) Metatibial spurs. We provide the first description of the metatibial spurs of A. bruchi, which show a distinctive morphology in the genus *Anillidris*, male and queen (Figure 5B and Figure 11C). In fact, they differ in shape, length, and cuticular fringes from the metatibial spurs of other dolichoderine genera, where they are thin and long with short cuticular fringes on the outer and inner margins [12];

(f) Pretarsal claws. We made the first description of the pretarsal claws that present a distinctively large base in males and queens; 

(g) Metapleural gland orifice. The orifice of the metapleural gland is absent in the male of *A. bruchi* (Figure 4A–D). Bolton [13], in the diagnosis of the family Formicidae, mentions the lack of the metapleural gland in most male species, but the presence of the gland and its morphology remain unknown in most male ants [14]. The absence of the metapleural gland is rarely found in worker and queen castes; in particular, it is absent in various species of the subfamily Formicinae [14,15]. Furthermore, in the aforementioned studies, it is highlighted that the absence of a metapleural gland orifice is found only in males of some species belonging to the subfamily Myrmeciinae, Dorylinae, Ponerinae, Pseudomyrmecinae, Aneuretinae, and Mirmicinae, but not in species of the subfamily Dolichoderinae. To our knowledge, in the available morphological descriptions of dolichoderine males, the absence of the metapleural gland orifice is not mentioned; therefore, *A. bruchi* currently represents the only species with a certain absence of the metapleural gland orifice in males of the subfamily Dolichoderinae. The description of the metapleural gland orifice in *A. bruchi* queen is also here supplied for the first time; 

(h) Wing venation. We provide a complete description of the wings with illustrations. In particular, we show for the first time the presence of nebulous remnants of the M2 vein distally, in the hindwing of the queen and male. It is worth noting that *Anillidris* shows complete venation in male forewings, a pattern present among the male Dolichoderinae only in *Linepithema* fuscum-group, *Dolichoderus*, *Leptomyrmex*, *Liometopum*, *Aptinoma*, and *Tapinoma* [6];

(i) Male external genitalia. Santschi [4] described the general shape of the male external genitalia with schematic drawings in lateral and ventral views. By using SEM, we described this complex part more accurately and highlighted some unknown characters. In particular, we emphasize that the basimere shows a ventral lobe (Figure 6A,B), the valviceps lamina presents an ondulate profile without distinct teeth (Figure 6D), and the volsella presents: (1) a reduced lateral cuspis (Figure 6C and Figure 7A–C); (2) a reduced basivolsellar process (Figure 6B and Figure 7B); and (3) a digitus apicodorsal process with spiniform sensilla in the posterolateral surface (Figure 7A–D). We assume that the spiniform sensilla in the digitus have a mechano-sensorial function during mating; they could be involved in clinging and perceiving the pressure exerted on the female genital tract. In fact, it has been observed in ants that the musculature of the volsella is well developed, indicating an important role for this part in facilitating the introduction of the penis valve [16,17,18,19]. In *A. bruchi* males, the cuspis is reduced, and the digitus probably acts as a dilator by using antagonistic volsellar muscles [19], spreading apart the membranes of the female and allowing access to the penis valve. We have called the cluster of mechano-sensorial modified sensilla on the digitus the “volsella sensorium”. 

Some modified setae on the posterolateral surface of the digitus were mentioned, for example, by Forbes et al. [20] and Yamada et al. [21], and in some descriptions of ant genitalia, it is possible to recognize in the figures such modified setae, but the authors did not describe them in detail and did not pay too much attention to these structures. Instead, we consider these modified setae as a peculiar functional cluster that we identify as a specific mechano-sensory area. We have observed the presence of this “volsella sensorium” in several species/genera of different ant subfamilies, with setae of different morphologies and positions (Cantone and Di Giulio pers. obs.). We hypothesize that the fine morphology and distribution of these setae can be important as both taxonomic and phylogenetic characters and deserve more in-depth investigations. 

## 5. Conclusions

In conclusion, in this study, using scanning electron microscopy (SEM), we described new morphological and morphometric distinctive features of the rare male and queen of *Anillidris bruchi* and redescribed them with more detail and using modern taxonomic standards [10,22,23], with attention to characters poorly described and illustrated in previous descriptions [2,4]. The results contribute to a more complete morphological diagnosis of the monotypic genus *Anillidris*.

## Data Availability

We will add images and specimens’ data to antweb.org (accessed on 7 July 2023).

## Supplementary Materials

The following supporting information can be downloaded at: https://www.mdpi.com/article/10.3390/insects14090723/s1, Figure S1: *Anillidris bruchi* forewing venation variation.
