# Supplementary figures and images for: A Fine Morphological Study of the Rare Anillidris bruchi Santschi (Hymenoptera: Formicidae: Dolichoderinae) Male and Queen
Source: Insects. 2023 Aug 23;14(9):723. doi: 10.3390/insects14090723 (PMC10532688; doi:10.3390/insects14090723)

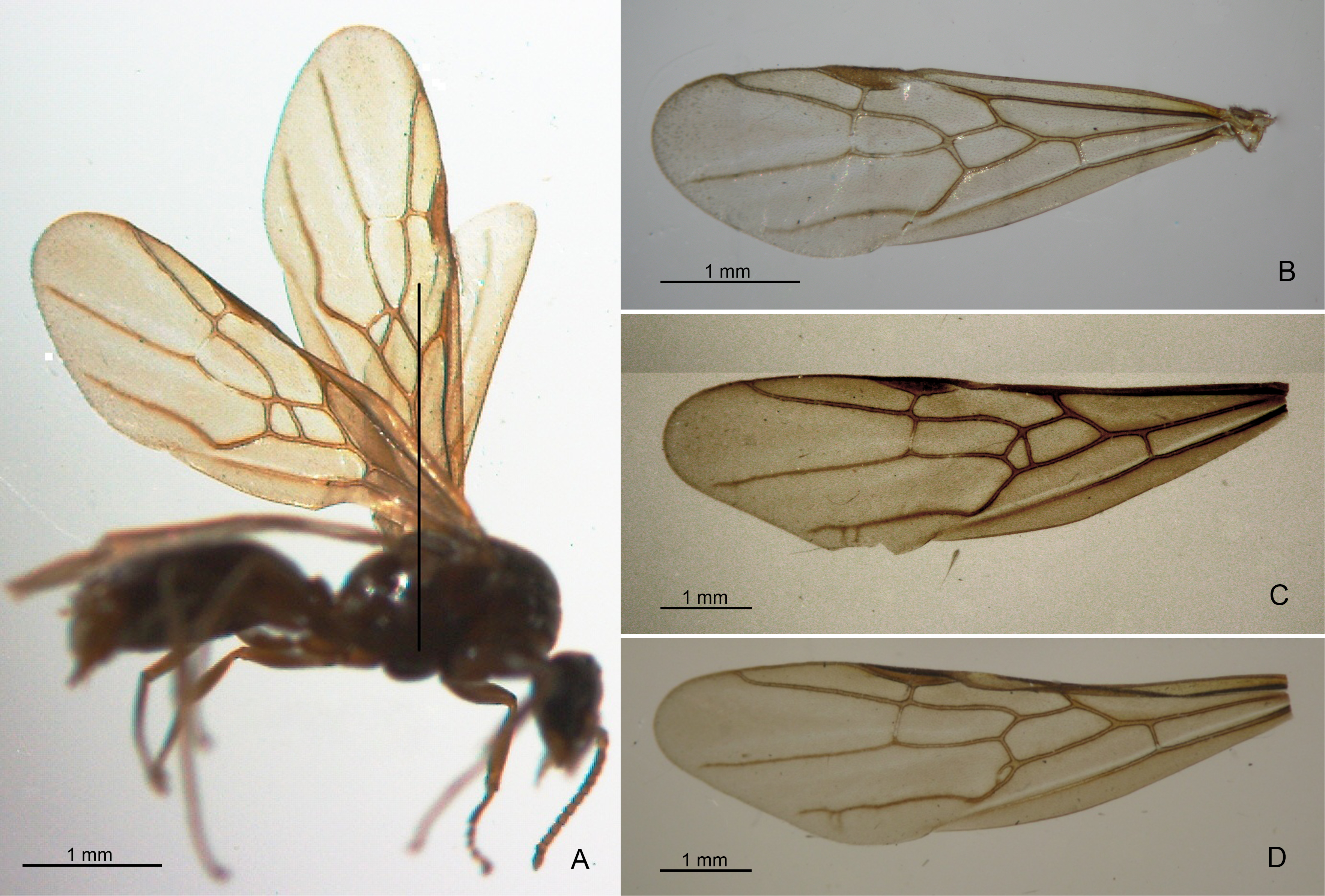

Supplement: Supplementary file 1 [file insects-14-00723-s001.zip › insects-2522776-supplementary.jpg]
